# The prediction value of platelet-derived growth factor for major adverse cardiovascular events in patients with acute non-ST-segment elevation myocardial infarction

**DOI:** 10.1080/07853890.2023.2176542

**Published:** 2023-03-13

**Authors:** Yan Liang, Jing-xian Wang, Xiao-Yuan Wu, Yan Cui, Zhong-He Zou, Wen-Qing Li, Yin Liu, Jing Gao

**Affiliations:** a Thoracic Clinical College, Tianjin Medical University, Tianjin, P.R. China; b Department of Cardiology, Tianjin Chest Hospital, Tianjin, P.R. China; c Cardiovascular Institute, Tianjin Chest Hospital, Tianjin, P.R. China; d Tianjin Key Laboratory of Cardiovascular Emergency and Critical Care, Tianjin Municipal Science and Technology Bureau, Tianjin, P.R. China

**Keywords:** Platelet-derived growth factor, non-ST-segment elevation myocardial infarction, major adverse cardiovascular events, prognosis

## Abstract

**Background:**

The value of plasma Platelet-Derived Growth Factor (PDGF) as a biomarker in predicting major adverse cardiovascular events (MACEs) in patients with acute non-ST-segment elevation myocardial infarction (NSTEMI) remains unclear.

**Methods:**

A total of 242 patients with NSTEMI were enrolled in this observational cohort study. The correlation between PDGF and MACEs was evaluated during a five-year follow-up. Kaplan–Meier survival analysis with Cox proportional-hazards regression was used to identify predictive values of PDGF.

**Results:**

The mean follow-up of NSTEMI patients was 1334 days. It was found that as the PDGF level increased, a significant uptrend in the incidence of MACEs and all-cause death, including the MACEs of 30 days, 180 days, 1 year, 5 years and the death of 1 year and 5 years (All Log-rank *p* < .05). Subgroup analysis further showed that PDGF had better predictive value for patients with age >65 years, GRACE score ≥140 and platelet count (PLT) >200 × 10^9^/L.

**Conclusion:**

PDGF levels can predict short-term and long-term MACEs in NSTEMI patients after discharge, especially for patients with older age, higher GRACE score and baseline PLT > 200 × 10^9^/L.

**Key messages**
- PDGF is a risk factor for short- and long-term MACEs in patients with STEMI.
- PDGF has a better prognostic value in patients with older age and PLT > 200 × 10^9^/L.
- Baseline plasma PDGF levels were positively correlated with GRACE score.

## Introduction

Myocardial infarction (MI) is a global public health problem contributing to high morbidity worldwide [1]. It is estimated that more than 7 million people in the world are diagnosed with MI each year. Among them, about 70% of patients with MI presented without significant ST-segment elevation on electrocardiography (ECG), which is known as non-ST-segment elevation MI (NSTEMI) [2]. Although mortality during the acute phase of NSTEMI is somewhat lower than that in STEMI, the long-term mortality is similar [3]. Therefore, it is of great importance to identify the potential risk factors of major adverse cardiovascular events (MACEs) in NSTEMI patients after discharge, which can help improve the prognosis and realize the individualized management of patients.

In recent years, great advances have been made in the understanding of the molecular biology associated with MI, leading to faster diagnosis, improved staging and timely treatment of patients [4]. Platelet-derived growth factor (PDGF), a potent mitogen and chemo-attractant for vascular smooth muscle cells (SMCs), is mainly derived from platelets, but is also exists in damaged endothelial cells, fibroblasts, macrophages and mesangial cells [5]. Matrix metalloproteinase (MMP), which is functionally related to PDGF, is involved in the degradation of extracellular matrix proteins, leading to the migration of SMCs to the intima and the rupture of plaques [6]. PDGF is inactive in monomeric form and exerts biological functions when specifically binds to its receptor (platelet-derived growth factor receptor, PDGF-R). The binding of PDGF to PDGFR can promote the replication and migration of myofibroblasts and participate in the occurrence and development of fibrotic diseases [7].

The main purpose of our study was to realize the change trend of serum PDGF in NSTEMI patients and to further clarify the prognostic role of PDGF in patients with NSTEMI after discharge.

## Materials and methods

### Study population

A total of 242 consecutive Chinese patients with NSTEMI were enrolled from January 2017 to December 2018. The inclusion criteria were as follows: (1) Patients diagnosed with NSTEMI. (2) The onset time was less than 28 days; (3) Coronary angiography and PCI were performed within 72 h of admission. The diagnostic criteria for NSTEMI are based on the European Society of Cardiology (ESC)/American College of Cardiology (ACC) NSTEMI guidelines: The disease history, physical examination and 12-lead ECG were evaluated by two cardiologists. Patients have high-sensitive cardiac troponin T (hs-cTnT) values > 99th upper limit of normal (ULN) and/or CK-MB >99th ULN, accompanied by one or more of the following conditions: (1) ST-segment depression and/or T-wave inversion on ECG; (2) Persistent chest pain for more than 30 min [8,9]. The exclusion criteria were as follows: (1) Persistent ST-segment elevation or chest pain caused by other cardiac disease or non-cardiac causes, such as myocarditis, aortic dissection, pulmonary embolism, etc.; (2) complicated with other severe diseases, such as severe liver or kidney dysfunction, severe bleeding, severe thrombocytopenia, stroke within a month, etc.; (3) cognitive or communication disorders; (4) pregnant woman; (5) patients who refuse to sign the informed consent.

All patients agreed to participate in the study after being informed of the procedures and risks involved. The study was conducted in accordance with the declaration of Helsinki, and the study protocol was approved by the ethical committee of Tianjin Chest Hospital. Some patients were enrolled from the Tianjin Inpatient Acute Myocardial Infarction Registry (TAMI) registered at ClinicalTrials.gov (identifier NCT03600259).

### Data collection

Baseline demographic and clinical information collected in our study included age, gender, vital signs, disease history, comorbidities, medications, laboratory indicators, etc. The Global Registry of Acute Coronary Events (GRACE) risk score was calculated on admission using eight variables, including age, heart rate, systolic blood pressure (SBP), Killip classification, serum creatinine, the presence of cardiac arrest, elevated biomarkers of MI and ST segment changes.

Fasting blood was collected the next morning after admission. All blood samples were temporarily stored at 4 °C. Within 2 h, serum was obtained by centrifugation at 3,000 rpm for 10 min and then frozen and stored at −80 °C until measurement. Plasma PDGF levels were detected by ELISA using a commercial kit (R&D Systems, Minneapolis, United States).

### Follow-up and outcome assessment

Clinical follow-up was scheduled at 30 ± 10 days after discharge and 6 ± 1, 12 ± 1 and 60 ± 3 months after discharge. The follow-up methods included readmission, myocardial infarction follow-up clinic visits and telephone contact.

The outcome was composite MACEs, that include all-cause death, angina uncontrollable by medication, non-fatal recurrent MI, hospital admission for heart failure and target lesion revascularization (TLR). Non-fatal MI was defined as a typical angina attack lasting more than 20 min, dynamic evolution on ECG and hs-cTnT > 99^th^ ULN and/or CK-MB >99^th^ ULN. Heart failure was diagnosed according to ESC guidelines [10]. TLR was defined as clinically driven coronary revascularization due to restenosis.

### Statistical analysis

Categorical variables were expressed as counts and percentages, and differences between groups were compared using the Pearson Chi-square test or Fisher’s exact test. Continuous variables were presented as mean ± standard deviation (SD) or median with interquartile range when data were not normally distributed. Differences between groups were compared using student’s t-test, one-way analysis of variance (ANOVA) and Kruskal–Wallis H test. Spearman correlation analysis was used to examine the association between PDGF and other biomarkers. Kaplan–Meier curve was used to estimate the event-free survival, and log-rank test was used to compare the curves between groups. Cox regression was used for subgroup analysis. All analyses were performed using SPSS software (Version 26.0; IBM, Munich, Germany). A *p* value <.05 was considered statistically significant.

## Results

### Cut-off point selection

In order to achieve the homogeneity of survival within the group and heterogeneity between groups, plasma PDGF levels were divided into three groups by using the X-tile (Yale University, New Haven, CT, United States), a common tool for obtaining optimal cut points based on biomarker expression [11,12].

As shown in Table 1 and Supplementary Figure 1, patients in the study were classified into three groups according to serum PDGF levels: low (114.96 − 421.48 ng/ml), medium (421.48 − 616.47 ng/ml) and high (616.47 − 947.76 ng/ml). The proportions of patients with MACE in each group were 10.37%, 16.46% and 32.14%, respectively.

**Table 1.** Cut-off point of PDGF (ng/mL) by X-tile method.

Group	Range (ng/ml)	Patient	Percentage (%)	MACEs in 1 year (%)	MACEs in 5 years (%)
Low	114.96–421.48	135	55.79	14(10.37)	36 (26.7)
Medium	421.48–616.47	79	32.64	13(16.46)	34 (43.0)
High	616.47–947.76	28	11.57	9(32.14)	13 (46.4)
Total	114.96–947.76	242	100	36(14.88)	83 (34.3)

### Baseline characteristics

Table 2 shows the baseline characteristics of 242 NSTEMI patients grouped by serum PDGF levels. The mean age of the study cohort was 63 ± 12 years, of whom 173 were males (71.49%). Mean systolic and diastolic pressures were 134.25 ± 21.23 mmHg and 77.67 ± 13.32 mmHg, respectively. Hyperlipidaemia was the most common comorbidity (78.2%), followed by hypertension (65.3%). In terms of lifestyle habits, more than half of the patients had a history of smoking, which is considered a high-risk factor for coronary artery disease. Most patients (98.8%) received dual antiplatelet therapy (DAPT) postoperatively, but the proportion of patients in the high PDGF group was significantly lower than that of the other two groups (*p* = .036). There were no significant differences between groups in transthoracic echocardiography (TTE) parameters, number of coronary lesions and intervention details (All *p* > .05).

**Table 2.** Baseline characteristics of the patients with NSTEMI in different PDGF levels.

Characteristics	Overall (*n* = 242)	Low (*n* = 135)	Medium (*n* = 79)	High (*n* = 28)	*p* Value
Age, years	63.71 ± 12.21	61.73 ± 11.79	67.10 ± 11.69	63.71 ± 13.86	.008
Male, n (%)	173(71.5)	101(74.8)	52(65.8)	20(71.4)	.372
Systolic pressure, mmHg	134.25 ± 21.23	134.47 ± 21.98	134.72 ± 21.66	131.89 ± 16.23	.821
Diastolic pressure, mmHg	77.67 ± 13.32	77.74 ± 12.91	77.28 ± 14.04	78.39 ± 13.64	.927
Heart rate, bpm	75.22 ± 15.54	76.07 ± 16.25	73.47 ± 15.06	76.07 ± 13.31	.475
IABP, n (%)	13(5.4)	6(4.4)	6(7.6)	1(3.6)	.594
Breathing machine, n (%)	32(13.2)	19(14.1)	11(13.9)	2(7.1)	.600
History, n (%)					
Smoking	139(57.4)	82(60.7)	42(53.2)	15(53.6)	.506
Drinking	62(25.6)	35(25.9)	21(26.6)	6(21.4)	.859
Hypertension	158(65.3)	83(61.5)	54(68.4)	21(75.0)	.308
Diabetes	82(33.9)	51(37.8)	23(29.1)	8(28.6)	.355
Stroke	51(21.1)	24(17.8)	19(24.1)	8(28.6)	.325
Hyperlipidaemia	186(78.2)	107(80.5)	59(75.6)	20(74.1)	.618
CHD	27(11.2)	18(13.3)	8(10.1)	1(3.6)	.308
Previous MI	34(14.0)	19(14.1)	11(13.9)	4(14.3)	.999
Biochemical characteristics, median (Q1, Q3)					
MCV, fl	89.80(87.00,92.55)	89.80(87.20,92.25)	90.35(86.28,93.43)	92.50(88.28,94.53)	.042
MCHC, g/l	340.00(332.00,346.00)	341.00(333.00,346.50)	339.00(332.75,345.00)	335.50(327.75,342.50)	.041
Lymphocyte count, 10^9^/L	1.70(1.21,2.17)	1.76(1.30,2.19)	1.50(1.06,2.04)	2.08(1.57,2.53)	.002
PLT, 10^9^/L	207.50(176.00,253.00)	213.00(181.50,266.50)	204.00(175.75,231.25)	202.00(172.75,238.75)	.014
MPV, fl	10.10(9.40,10.80)	10.10(9.10,10.60)	10.30(9.70,11.33)	10.40(9.68,11.65)	.013
P-LCR, %	27.70(23.35,33.23)	26.60(22.60,30.90)	28.70(24.90,35.20)	28.80(25.10,37.40)	.016
NT-proBNP, pg/ml	868.10(324.13,2385.75)	671.60(234.40,2237.00)	1108.00(368.68,2959.50)	939.10(397.25,3279.75)	.039
Hs-cTnT, ng/ml	0.55(0.28,1.29)	0.51(0.22,1.27)	0.41(0.26,1.48)	0.73(0.34,1.40)	.257
CK, U/L	229.50(104.75,587.00)	246.00(101.00,808.00)	233.50(107.50,544.25)	166.50(88.25,381.00)	.803
CK-MB, U/L	29.00(17.00,68.00)	29.00(17.00,85.00)	30.00(19.00,64.00)	23.00(15.75,49.25)	.685
TC, mmol/l	4.58(3.78,5.19)	4.66(3.86,5.14)	4.62(3.70,5.28)	4.54(3.40,4.96)	.604
LDL-C, mmol/l	3.05(2.30,3.59)	2.94(2.28,3.51)	3.04(2.23,3.68)	2.80(2.10,3.53)	.509
D-dimer, µg/ml	0.43(0.29,0.74)	0.37(0.26,0.60)	0.52(0.34,0.93)	0.40(0.27,0.70)	.040
hs-CRP, mg/l	5.06(2.43,15.38)	4.71(2.53,12.64)	5.48(1.89,19.49)	6.30(3.52,16.93)	.800
Medication during follow-up, n (%)					
DAPT	239(98.8)	134(99.3)	79(100)	26(92.9)	.036
Beta-blocker	202(83.5)	114(84.4)	67(84.8)	21(75.0)	.438
ACEI/ARB	148(61.2)	81(60.0)	50(63.3)	17(60.7)	.891
Statin	236(97.5)	130(96.3)	79(100.0)	27(96.4)	.161
Anticoagulant	241(99.6)	134(99.3)	79(100.0)	28(100.0)	1.000
Coronary lesion segment, n (%)					
Single lesion	45(18.6)	21(19.3)	15(23.1)	9(36.0)	.195
Double lesions	37(15.3)	23(21.1)	10(15.4)	4(16.0)	.605
Three lesions	114(47.1)	64(58.7)	39(60.0)	11(44.0)	.352
Left main-vessel lesion	27(11.2)	11(10.1)	10(15.4)	6(24.0)	.163
Stents	2(1,6)	1(1,3)	2(1,6)	2(1,3)	.464
Killip classification, n (%)					
I	202(83.5)	117(86.7)	61(77.2)	24(85.7)	
II-IV	40(16.5)	18(13.3)	18(22.8)	4(14.3)	
GRACE score	126.03 ± 38.91	121.41 ± 38.62	136.22 ± 35.52	133.93 ± 40.17	.015
＜109	82(33.9)	53(39.3)	21(26.6)	8(28.6)	
109-140	82(33.9)	55(40.7)	21(26.6)	6(21.4)	
>140	78(32.2)	27(20.0)	37(46.8)	14(50.0)	
Transthoracic echocardiography, M ± SD					
LAD, mm	39.13 ± 5.01	39.27 ± 5.12	39.38 ± 5.27	37.81 ± 3.52	.345
LVEDD, mm	52.07 ± 5.96	52.03 ± 6.26	51.76 ± 5.43	53.11 ± 6.05	.602
LVEF, n (%)	51.47 ± 8.39	51.49 ± 8.58	51.90 ± 8.06	50.19 ± 8.63	.664
Outcome in 1 year, n (%)					
Composite MACEs	36(14.9)	14(10.4)	13(16.5)	9(32.1)	.012
All-cause death	8(3.3)	2(1.5)	3(3.8)	3(10.7)	.043
Non-fatal recurrent MI	8(3.3)	2(1.5)	5(6.3)	1(3.6)	.149
Target lesion revascularization	3(1.2)	1(0.7)	1(1.3)	1(3.6)	.344
Hospital admission for HF	11(4.5)	7(5.2)	2(2.5)	2(7.1)	.395
Angina pectoris that can’t be controlled by medication	11(4.5)	4(3.0)	4(5.1)	3(10.7)	.133

*Note:* IABP: intra-aortic balloon pump; CHD: coronary heart disease; MCHC: mean red blood cell haemoglobin concentration; PLT: platelet count; MPV: mean platelet volume; P-LCR: platelet -larger cell ratio; NT-proBNP: N-terminal pro-B type natriuretic peptide; hs-cTnT: hypersensitive troponin T; CK: creatine kinase; TC: total cholesterol; HDL-C: high-density lipoprotein cholesterol; LDL-C: low-density lipoprotein cholesterol; hs-CRP: high-sensitivity C-reactive protein; DAPT: dual antiplatelet therapy; ACEI: angiotensin-converting enzyme inhibitors; ARB: angiotensin II receptor blockers; GRACE: Global Registry of Acute Coronary Events; LAD: left atrial diameter; LVED: left ventricular end diastolic diameter; LVEF: left ventricular ejection fraction; MI: myocardial infarction; MACE: major adverse cardiovascular event.

Table 3 shows the patients’ details stratified by MACEs within one year after discharge. Patients with MACEs were older (71.94 ± 9.61 vs 62.28 ± 12.06, *p* < .001) and had a faster heart rate (86.36 ± 20.44 vs 73.28 ± 13.68, *p* < .001), which may be caused by poor heart rate control. In addition, patients with MACEs had significantly higher GRACE score (158.33 ± 40.43 vs 120.39 ± 35.86, *p* < .001), lower left ventricular ejection fraction (LVEF) (47.72 ± 8.20 vs 52.11 ± 8.28, *p* = .002) and lower Killip classification (Killip II-IV: 41.7% vs 12.1%, *p* < .001). N-terminal pro-B type natriuretic peptide (NT-proBNP) and high sensitivity C-reactive protein (hsCRP) levels were significantly higher in patients with MACEs (all *p* < .001). In addition, serum PDGF levels were higher in patients who experienced MACEs (*p* = .027).

**Table 3.** Baseline characteristics of the patients stratified by MACEs.

	Non-MACE (*N* = 206)	MACE (*N* = 36)	*p* Value
Age (years)	62.28 ± 12.06	71.94 ± 9.61	<.001
Male, n(%)	152(73.9)	21(58.3)	.058
Systolic pressure, mmHg	134.84 ± 21.00	130.89 ± 22.48	.304
Diastolic pressure, mmHg	78.36 ± 13.14	73.78 ± 13.85	.057
Heart rate	73.28 ± 13.68	86.36 ± 20.44	<.001
Smoking	122(59.2)	17(47.2)	.179
Hypertension	133(64.6)	25(69.4)	.570
Diabetes	66(32.0)	16(44.4)	.147
Hyperlipidaemia	164(80.0)	22(66.7)	.085
DAPT	204(99.0)	35(97.2)	.385
Beta-blocker	174(84.5)	28(77.8)	.319
ACEI/ARB	128(62.1)	20(55.6)	.455
Statin	202(98.1)	34(94.4)	.198
LVEF	52.11 ± 8.28	47.72 ± 8.20	.002
Killip classification, n (%)	<.001		
I	181(87.9)	21(58.3)	
II-IV	25(12.1)	15(41.7)	
GRACE score	120.39 ± 35.86	158.33 ± 40.43	<.001
<109	78(37.9)	4(11.1)	
109–140	74(35.9)	8(22.2)	
>140	54(26.2)	24(66.7)	
hsTnT, ng/ml	0.52(0.24,1.29)	1.05(0.46,2.08)	.081
NT-proBNP, pg/ml	702.90(312.10,2163.00)	3349.00(1081.50,5413.25)	<.001
CK, U/L	246.00(103.00,683.00)	345.50(126.75,1367.25)	.359
CK-MB, U/L	30.00(17.00,74.00)	33.50(19.50,101.75)	.713
TC, mmol/l	4.61(3.86,5.28)	4.32(3.41,4.95)	.165
LDL-C, mmol/l	2.87(2.18,3.55)	3.07(3.42,3.87)	.264
D-dimer, µg/ml	0.43(0.28,0.66)	0.59(0.32,1.32)	.045
hs-CRP, mg/l	4.64(2.00,12.03)	10.13(4.33,59.10)	<.001
PLT, 10^9^/L	208.00(177.50,253.00)	199.00(172.00,256.00)	.061
PDGF, ng/ml	396.19(304.42,504.73)	512.25(334.37,618.26)	.027

*Note:* MACE: major adverse cardiovascular event; DAPT: dual antiplatelet therapy; ACEI: angiotensin-converting enzyme inhibitors; ARB: angiotensin II receptor blockers; LVEF: left ventricular ejection fraction; GRACE: Global Registry of Acute Coronary Events; NT-proBNP: N-terminal pro-B type natriuretic peptide; hsTNT: hypersensitive troponin T; CK: creatine kinase; TC: total cholesterol; HDL-C: high-density lipoprotein cholesterol; LDL-C: low-density lipoprotein cholesterol; hs-CRP: high-sensitivity C-reactive protein; PLT: platelet count; PDGF: platelet-derived growth factor.

### Correlation of PDGF with other biomarkers and GRACE score

**Figure 1.**
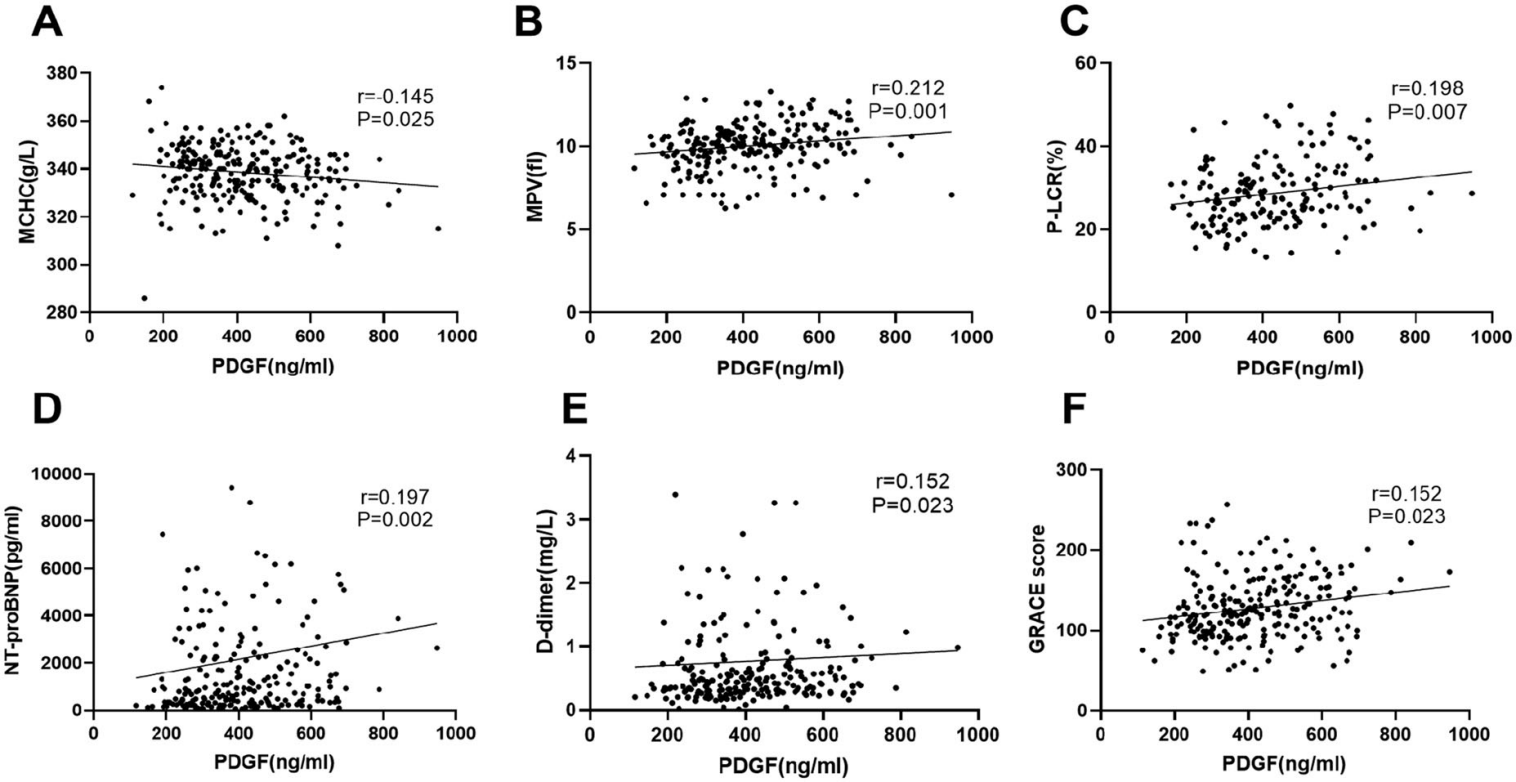
Correlation between plasma PDGF levels and other biomarkers. Panels A–F represent the correlation between plasma PDGF levels and mean cell haemoglobin concentration (MCHC), mean platelet volume (MPV), platelet-larger cell ratio (P-LCR), D-dimer, N-terminal pro-B type natriuretic peptide (NT-proBNP), and hypersensitive troponin T (hs-cTNT), respectively.

Table 2 shows several biological indicators related to PDGF levels. Spearman rank correlation analysis was performed to examine the correlation of PDGF with other biomarkers and GRACE score (Figure 1). PDGF levels were significantly correlated with MCHC (*r* = 0.145, *p* = .025), MPV (*r* = 0.212, *p* = .001), D-Dimer (*r* = 0.152, *p* = .023), platelet-larger cell ratio (P-LCR) (*r* = 0.198, *p* = .007), NT-proBNP (*r* = 0.197, *p* = .002) and GRACE score (*r* = 0.152, *p* = .023). After grouping by GRACE score, the higher the GRACE score, the higher the PDGF level (All *p* < .05 between groups) (Figure 3).

### Follow-up and results

All patients were followed up for 5 years, with a median follow-up time of 1334 days. During the one-year follow-up period, 36 (14.9%) patients experienced at least one MACE, including 8 cases of all-cause death (3.3%), 8 cases of non-fatal recurrent MI (3.3%), 3 cases of target lesion revascularization (1.2%), 11 cases of hospitalization due to heart failure (4.5%) and 11 cases of angina pectoris that were not well-controlled by medication (4.5%). There were 14 (10.4%), 13 (16.5%) and 9 (32.1%) patients experienced MACE in the low, medium and high levels of PDGF groups, respectively. There were two (1.5%), three (3.8%), three (10.7%) patients died, respectively. There were significant differences in MACE (*p* = .012) and mortality (*p* = .043) among the three groups. During the five-year follow-up period, 83 patients have experienced MACE, of which 19 patients died (Table 1 and Table 2).

**Figure 2.**
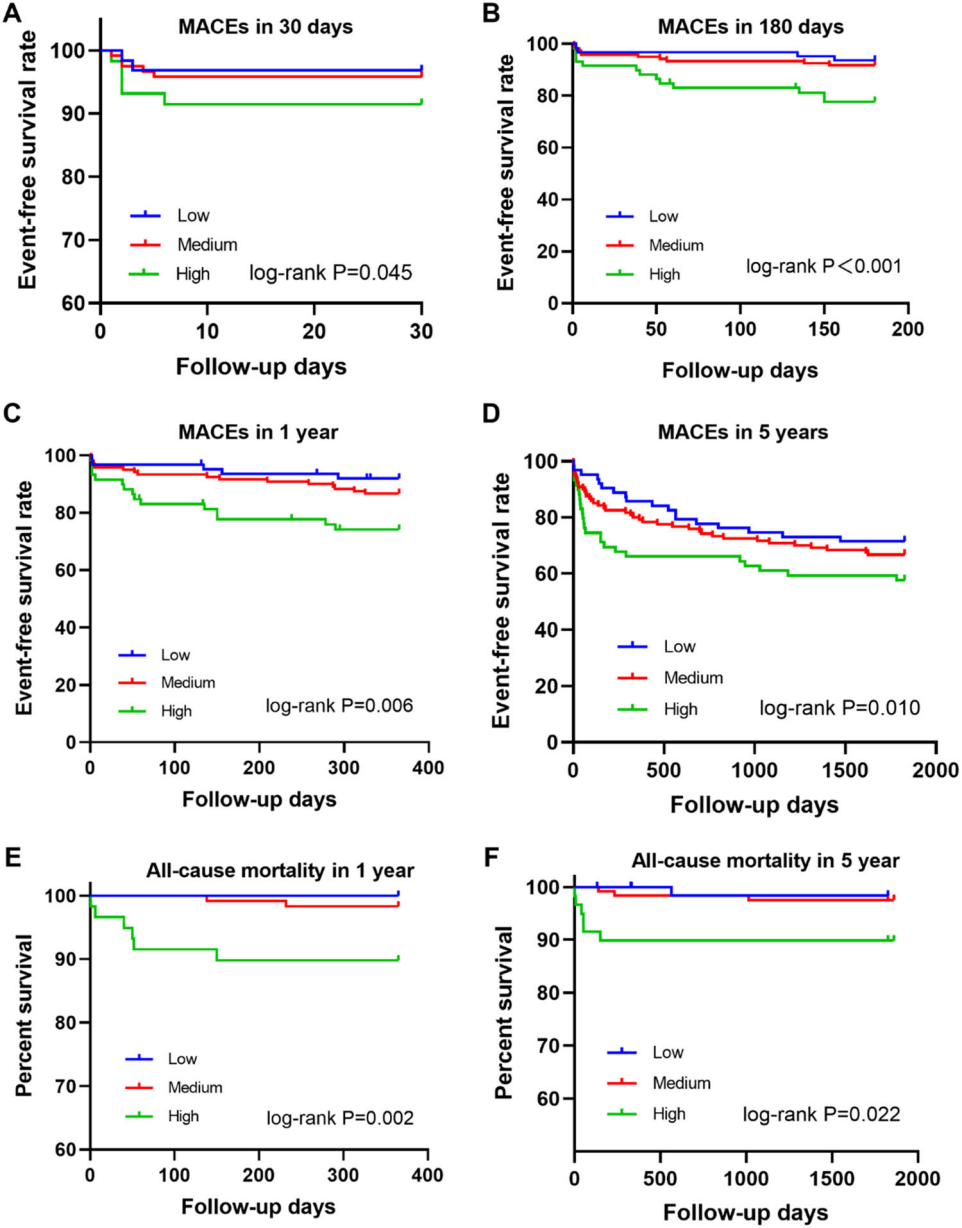
Kaplan–Meier survival curves of MACEs in 242 NSTEMI patients. (A) 30-day MACEs. (B) 180-day MACEs. (C) 1-year MACEs. (D) 5-year MACEs. (E) 1-year all-cause mortality. (F) 5-year all-cause mortality.

As shown in Figure 2, PDGF levels were significantly associated with short- and long-term MACEs. Compared with patients with low and medium PDGF levels, patients with high PDGF levels had lower, 30-day, 180-day, 1-year and 5-year event-free survival rate and lower 1-year and 5-year mortality (*p* < .05).

**Figure 3.**
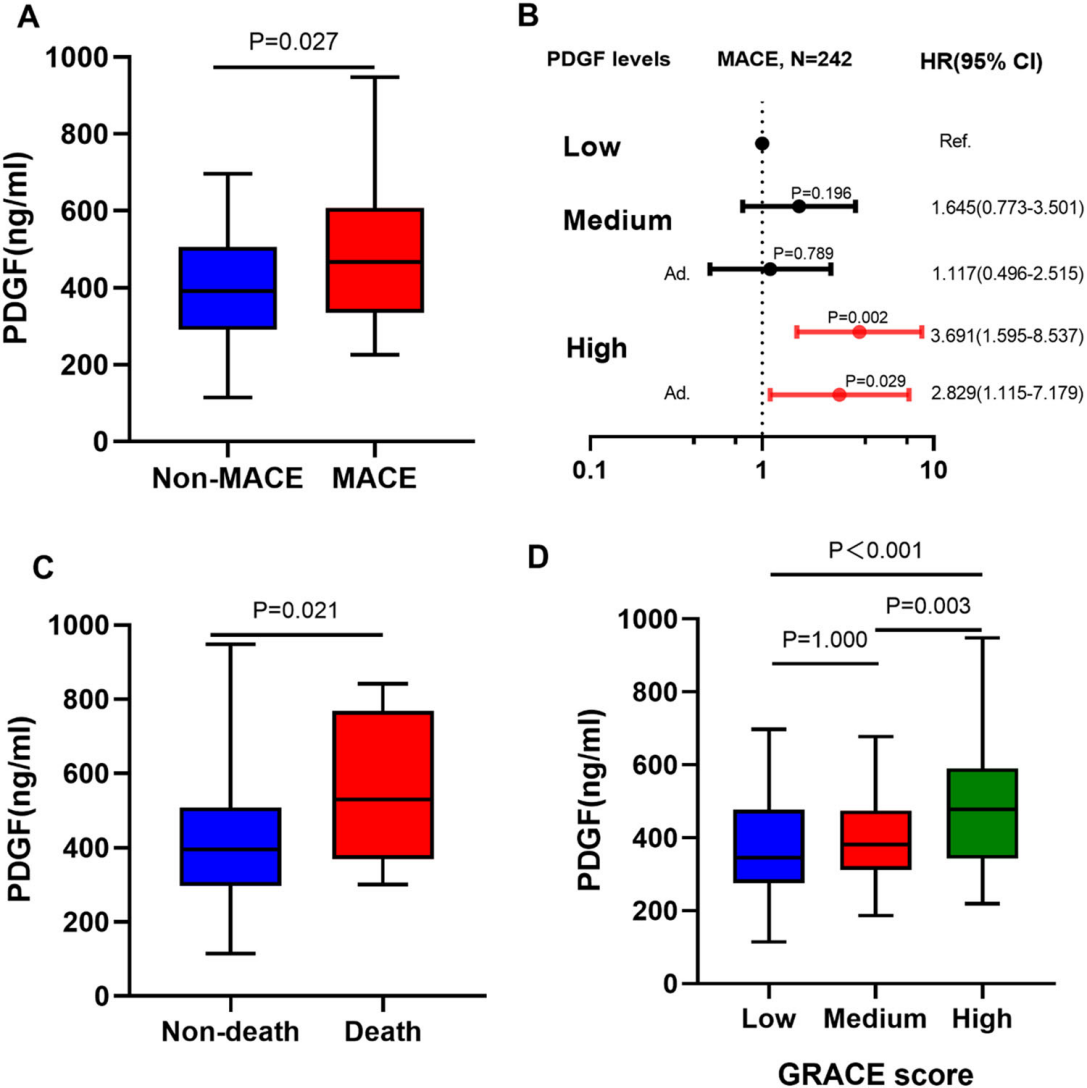
(A) Comparison of plasma PDGF levels between groups based on MACEs. (B) Forest plot shows the HRs with 95% CIs for MACE with regard to age, sex, smoking, hypertension, diabetes, stroke, hyperlipidaemia, previous MI and family history of CHD. (C) Comparison of plasma PDGF levels between groups based on all-cause death. (D) Comparison of plasma PDGF levels between groups based on GRACE score.

After adjusting age, sex, smoking, hypertension, diabetes, stroke, hyperlipidaemia, previous MI and family history of coronary heart disease, patients with high PDGF levels had a higher risk of MACE within one year than those with low PDGF levels (HR = 2.829, 95% CI: 1.115 − 7.179; *p* = .029). Similar results were observed at 6-month (HR = 4.235, 95% CI: 1.503 − 11.933; *p* = .006) and five-year follow-up (HR = 2.225, 95% CI: 1.130 − 4.384; *p* = .021) (Figure 3 and Supplementary Table 1).

**Figure 4.**
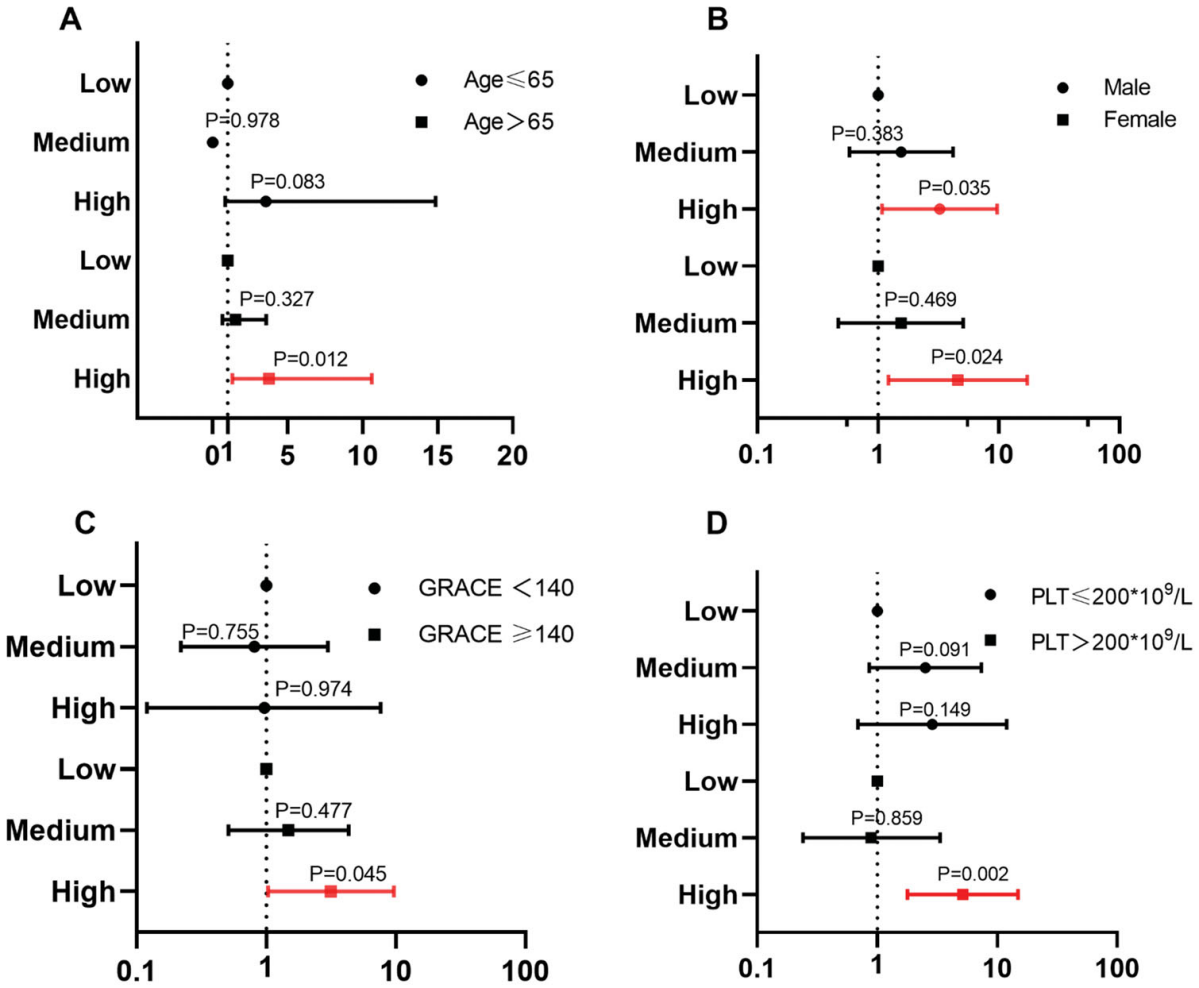
Subgroup analysis of different patients. Panels A–D represent the subgroup analyses stratified by ages, NT-proBNP levels, GRACE score and PLT level.

Multivariate Cox regression models were further used for subgroup analysis of composite MACEs at different PDGF levels. As shown in Figure 4 (detailed results are displayed in Supplementary Table 2), the predictive value was more significant for patients with age > 65 years (high PDGF level vs. low level, adjusted HR = 3.76, 95% CI: 1.33 − 10.61; *p* = .012), GRACE score ≥ 140 (high PDGF level vs. low level, adjusted HR = 3.16, 95% CI: 1.03 − 9.68, *p* = .045),and PLT > 200 × 10^9^/L (high PDGF level vs. low level, adjusted HR = 5.16, 95% CI: 1.78 − 14.94; *p* = .002). There was no statistically significant difference in the predictive value of PDGF levels between males and females.

## Discussion

Our study explored the association between plasma PDGF levels and prognosis in patients with NSTEMI. Three main findings were as follows: First, PDGF is a risk factor for short- and long-term MACEs in NSTEMI patients after discharge. Second, the prognostic utility of PDGF was more obvious for NSTEMI patients > 65 years and with a PLT > 200 × 10^9^/L. Third, plasma PDGF levels were positively correlated with baseline GRACE scores, which may contribute to effective risk stratification in patients with NSTEMI.

As one of the growth factors, PDGF is a glycoprotein composed of four different polypeptide chains. After protein translation, these chains form disulphide-bonded dimers that function as a biologically active growth factor [13]. PDGF is known to be a powerful inducer of cell mitosis, migration, angiogenesis and matrix regulation and plays a key role in cardiac tissue development, homeostasis and healing [14].

Atherosclerotic lesions are closely related to chronic inflammation. A recent systematic review by Kurtul et al. [15] found that the platelet-to-lymphocyte ratio is closely associated with cardiovascular disease (CVD) and can be used as an inflammatory marker to predict prognosis in different types of CVD. PDGF is released by different kinds of immune cells and acts on SMCs on the vascular wall. PDGF contributes to neointimal thickening, an early sign of atherosclerosis [16]. Serum PDGF-BB is a key mediator of arterial stiffness in response to ageing and metabolic stress and can be used as a biomarker to predict the risk of age-related vascular disease [17].

In recent years, many studies have confirmed that a variety of cytokines, including PDGF, can participate in the pathogenesis of ACS through different pathophysiological pathways. Koizumi et al. [6] found that PDGF-BB plays a role in coronary plaque instability in the acute phase of STEMI. Furthermore, serum PDGF-BB levels correlated with ischaemic time and CK-MB levels may predict the degree of cardiomyocyte ischaemic injury [18]. Another study by Alehagen et al. also found that PDGF-D levels were increased in infarcted tissue [19]. A recent clinical study [5] showed that the serum PDGF concentration in ACS patients was significantly higher than that in the non-coronary heart disease group, and the serum PDGF concentration in patients with acute MI was significantly higher than that in patients with unstable angina.

The results of the present study suggest that serum PDGF may play a potential role in the pathogenesis of MI. The possible molecular mechanism is as follows: PDGF is a principal survival factor that inhibits apoptosis and promotes proliferation. During the acute phase of MI, the communication between endothelial cells and cardiomyocytes may induce more PDGF production, thereby preventing cardiomyocyte apoptosis [18]. Meanwhile, platelet aggregation occurs immediately after vascular injury in the injured area and activated platelets release a large amount of PDGF. The results of our study found that PDGF levels correlated with platelet and D-dimer levels, further supporting the assumption of the above possible molecular mechanism.

PDGF is a key mediator of fibrosis, and blocking its binding to its receptor reduces collagen content in infarcts and may enhance ventricular remodelling. Deletion of PDGF-B or its predominant receptor gene PDGFR-β results in cardiac abnormalities, including late embryonic ventricular dilation, myocardial trabecular hyperplasia and septal abnormalities [20]. The previous study [21] has shown that the PDGF-BB/PDGFR-β signalling pathway could prevent cardiomyocyte apoptosis through PI3K/Akt signalling, which helps protect cardiomyocytes, reduce infarct size and preserve systolic function. However, excessive activation of PDGF-β instead induces fibroblast overgrowth, collagen accumulation and cardiac fibrosis [22]. Furthermore, overexpression of PDGF-D in mouse hearts [23] leads to interstitial fibrosis and dilative cardiomyopathy, resulting in cardiac failure [17]. The results of our study found that PDGF concentration was significantly correlated with cardiac function indicators, including NT-proBNP, Killip classification and LVEF, suggesting that PDGF level is associated with the deterioration of cardiac function, which provides indirect evidence of the above conclusion.

Based on the pivotal role of PDGF in anti-apoptosis and vasculogenic effects, PDGF may be a novel treatment target for heart failure. Activation of PDGFR signalling attenuates post-infarction remodelling and may have important therapeutic implications for survivors of acute MI [24]. In the MI animal model by Hsieh et al. [22], local delivery of PDGF significantly improved LVEF and cardiac index and significantly reduced end-diastolic volume, suggesting that PDGF attenuated remodelling in treated animals. In the study by Cui et al. [25], dual gene therapy with bFGF and PDGF showed a marked angiogenic effect, with concomitant significant reduction in infarct size and improved cardiac function. The possible mechanisms are as follows: First, PDGF-induced activation of endothelial cells may promote angiogenesis, thereby increasing capillary density in the infarct border zone [26]. Second, PDGFR-β activation in mural cells may promote vascular maturation. Third, enhanced PDGFR-α signalling may improve the repair function of fibroblasts and promote organized collagenous scar formation, which is critical for protecting the ventricle from rupture and adverse remodelling [27].

The involvement of PDGF in the occurrence of MI and the subsequent cardiac remodelling implies an important predictive role of PDGF in the prognosis of MI patients. Zhang et al. [28] screened serum biomarker expression profiles of STEMI patients and found that PDGF could be used as a biomarker to predict the outcomes of patients with STEMI. In the study by Pang et al. [5], the mean PDGF levels in peripheral blood were higher in patients with major cardiovascular and cerebrovascular adverse events (MACCE) than in patients without MACCE. However, in the Kaplan–Meier curve analysis of 36-month MACCE, there was no significant difference in the risk of MACCE among the three groups stratified by PDGF levels in peripheral blood.

It is worth noting that the results of our study are not completely consistent with the above studies. In our study, serum PDGF levels in patients after MI were positively correlated with the development of MACE: the incidence of MACE increased with the increase of PDGF levels. The possible reason is that the site of severe infarction compensates for more PDGF production. On the other hand, our study further revealed the relationship between PDGF levels and GRACE score. In the Hung’s study [29], the GRACE score was used to estimate the risk of future all-cause mortality and MI. Hung et al. also found that patients with high GRACE score had high PDGF levels, suggesting that PDGF may be used as a biomarker for predicting adverse outcomes in patients with STEMI. The preliminary results of our study, together with previous studies, provide potential leads for further in-depth research and insight into NSTEMI healthcare. The clinical application of PDGF need to be further validated in large multicentre studies.

## Limitations

There are some limitations in our study. First, our study was conducted in a single centre with a limited number of patients. Therefore, the results of the present study may be influenced by confounding factors and cannot be extended to other races [30]. Second, medications taken by patients in the study cohort may include aspirin and clopidogrel, which may affect PDGF levels due to their antiplatelet effects. Third, our study did not further analyse the subtypes of PDGF, nor did it monitor the changes of PDGF during the follow-up period. Therefore, the effect of different PDGF subtypes and dynamic PDGF levels on MACEs remain uncertain. In addition, target lesion revascularization as an intervention always depends on the subjective judgement of the treating physicians. The same holds for rehospitalization and refractory angina symptoms, neither of which are clear dichotomous variables.

## Conclusions

Plasma PDGF is a potential biomarker for short- and long-term prognosis in NSTEMI patients after discharge, especially for those with older age, high GRACE risk score and baseline PLT > 200 × 10^9^/L.

## Supplementary Material

Supplemental Material

Supplemental Material

Supplemental Material

## Data Availability

All data used and analysed in the study are available by contacting the corresponding authors upon reasonable request.
