# Supplementary figures and images for: The prediction value of platelet-derived growth factor for major adverse cardiovascular events in patients with acute non-ST-segment elevation myocardial infarction
Source: Ann Med. 2023 Mar 13;55(1):1047–57. doi: 10.1080/07853890.2023.2176542 (PMC10795595; doi:10.1080/07853890.2023.2176542)

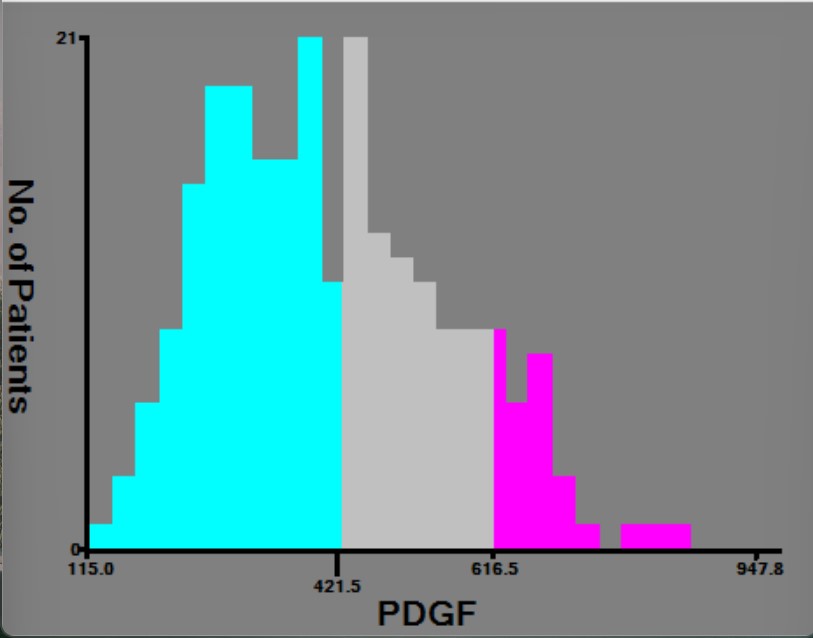

Supplement: Supplemental Material [file IANN_A_2176542_SM5274.jpg]
