# Supplementary material for: The prediction value of platelet-derived growth factor for major adverse cardiovascular events in patients with acute non-ST-segment elevation myocardial infarction
Source: Ann Med. 2023 Mar 13;55(1):1047–57. doi: 10.1080/07853890.2023.2176542 (PMC10795595; doi:10.1080/07853890.2023.2176542)
Supplement: Supplemental Material [file IANN_A_2176542_SM5264.docx]

Supplementary Table 2. Univariate and multivariate COX regression analysis of PDGF level in predicting MACE.

|  | **PDGF** | **HR (95% CI)** | **P-value** |
| --- | --- | --- | --- |
| **1 month MACEs (n=12)** | | | |
| Unadjusted | Low | - | - |
|  | Medium | 0.568(0.115-2.812) | 0.488 |
|  | High | 3.283(0.926-11.636) | 0.066 |
| Adjusted | Low | - | - |
|  | Medium | 0.345(0.065-1.822) | 0.210 |
|  | High | 1.837(0.391-8.638) | 0.441 |
| **6 months MACEs (n=27)** | | | |
| Unadjusted | Low | - | - |
|  | Medium | 1.748(0.694-4.403) | 0.236 |
|  | High | 5.455(2.163-13.757) | ＜0.001 |
| Adjusted | Low | - | - |
|  | Medium | 1.086(0.407-2.897) | 0.869 |
|  | High | 4.235(1.503-11.933) | 0.006 |
| **1 year MACEs (n=36)** | | | |
| Unadjusted | Low | - | - |
|  | Medium | 1.645(0.773-3.501) | 0.196 |
|  | High | 3.691(1.595-8.537) | 0.002 |
| Adjusted | Low | - | - |
|  | Medium | 1.117(0.496-2.515) | 0.789 |
|  | High | 2.829(1.115-7.179) | 0.029 |
| **5 years MACEs (n=83)** | | | |
| Unadjusted | Low | - | - |
|  | Medium | 1.815(1.136-2.901) | 0.013 |
|  | High | 2.176(1.154-4.105) | 0.016 |
| Adjusted | Low | - | - |
|  | Medium | 1.624(0.988-2.670) | 0.056 |
|  | High | 2.225(1.130-4.384) | 0.021 |

Adjusted age, sex, smoking, hypertension, diabetes, stroke, hyperlipidemia, previous MI and family history of CHD.
