# Supplementary material for: The prediction value of platelet-derived growth factor for major adverse cardiovascular events in patients with acute non-ST-segment elevation myocardial infarction
Source: Ann Med. 2023 Mar 13;55(1):1047–57. doi: 10.1080/07853890.2023.2176542 (PMC10795595; doi:10.1080/07853890.2023.2176542)
Supplement: Supplemental Material [file IANN_A_2176542_SM5255.docx]

Supplementary Table 1. Subgroup analysis of different patients.

| **Factors** | **PDGF** | **HR (95%CI)** | **P-value** |
| --- | --- | --- | --- |
| Age≤65 | Low | - | - |
|  | Medium | 0 | 0.978 |
|  | high | 3.55(0.85-14.86) | 0.083 |
| Age＞65 | Low | - | - |
|  | Medium | 1.53(0.65-3.58) | 0.327 |
|  | high | 3.76(1.33-10.61) | 0.012 |
| Male | Low | - | - |
|  | Medium | 1.55(0.58-4.17) | 0.383 |
|  | high | 3.24(1.08-9.67) | 0.035 |
| female | Low | - | - |
|  | Medium | 1.55(0.47-5.08) | 0.469 |
|  | high | 4.58(1.22-17.19) | 0.024 |
| GRACE score＜140 | Low | - | - |
|  | Medium | 0.81(0.22-3.00) | 0.755 |
|  | high | 0.966(0.12-7.63) | 0.974 |
| GRACE score≥140 | Low | - | - |
|  | Medium | 1.48(0.51-4.32) | 0.477 |
|  | high | 3.16(1.03-9.68) | 0.045 |
| PLT≤200*10^9^/L | Low | - | - |
|  | Medium | 2.52(0.86-7.38) | 0.091 |
|  | high | 2.87(0.69-12.02) | 0.149 |
| PLT＞200*10^9^/L | Low | - | - |
|  | Medium | 0.89(0.24-3.34) | 0.859 |
|  | high | 5.16(1.78-14.94) | 0.002 |

HR, Hazard ratio; GRACE, Global Registry of Acute Coronary Events; PLT, platelet count.
